# A Precision Health Service for Chronic Diseases: Development and Cohort Study Using Wearable Device, Machine Learning, and Deep Learning

**DOI:** 10.1109/JTEHM.2022.3207825

**Published:** 2022-09-19

**Authors:** Chia-Tung Wu, Ssu-Ming Wang, Yi-En Su, Tsung-Ting Hsieh, Pei-Chen Chen, Yu-Chieh Cheng, Tzu-Wei Tseng, Wei-Sheng Chang, Chang-Shinn Su, Lu-Cheng Kuo, Jung-Yien Chien, Feipei Lai

**Affiliations:** Department of Computer Science and Information EngineeringNational Taiwan University 33561 Taipei 10617 Taiwan; Graduate Institute of Biomedical Electronics and Bioinformatics, National Taiwan University 33561 Taipei 10617 Taiwan; Department of Internal MedicineCollege of MedicineNational Taiwan University Hospital, National Taiwan University 38006 Taipei 10617 Taiwan

**Keywords:** Precision health, artificial intelligence, wearable device, chronic obstructive pulmonary disease, panic disorder

## Abstract

This paper presents an integrated and scalable precision health service for health promotion and chronic disease prevention. Continuous real-time monitoring of lifestyle and environmental factors is implemented by integrating wearable devices, open environmental data, indoor air quality sensing devices, a location-based smartphone app, and an AI-assisted telecare platform. The AI-assisted telecare platform provided comprehensive insight into patients’ clinical, lifestyle, and environmental data, and generated reliable predictions of future acute exacerbation events. All data from 1,667 patients were collected prospectively during a 24-month follow-up period, resulting in the detection of 386 abnormal episodes. Machine learning algorithms and deep learning algorithms were used to train modular chronic disease models. The modular chronic disease prediction models that have passed external validation include obesity, panic disorder, and chronic obstructive pulmonary disease, with an average accuracy of 88.46%, a sensitivity of 75.6%, a specificity of 93.0%, and an F1 score of 79.8%. Compared with previous studies, we establish an effective way to collect lifestyle, life trajectory, and symptom records, as well as environmental factors, and improve the performance of the prediction model by adding objective comprehensive data and feature selection. Our results also demonstrate that lifestyle and environmental factors are highly correlated with patient health and have the potential to predict future abnormal events better than using only questionnaire data. Furthermore, we have constructed a cost-effective model that needs only a few features to support the prediction task, which is helpful for deploying real-world modular prediction models.

## I. Introduction

With the rapid progress of precision medicine, patients have the additional option of advanced and personalized medical treatment in hospitals. From a treatment point of view, this facilitates the selection of drugs that minimize side effects and produce the best results. However, from the perspective of prevention, many studies claim that numerous challenges remain in the field of precision medicine [1], [2]. Since precision medicine applications are developed based on historical electronic medical records, most treatments can be obtained only when the patient is hospitalized. The problem is that most people do not stay in the hospital for an extended period of time. Once a patient has been discharged from the hospital, there is a risk that lifestyle and environment will affect disease control and prevention. Hence, the broader concept of precision health has been proposed and continues to grow.

Precision health is defined as a holistic approach to help people stay healthy through personalized prevention and treatment, which focuses on the prevention of disease. This includes precision medicine, but with a greater emphasis on daily monitoring, health promotion, and disease prevention [3]. Several studies demonstrate the great potential of advancements in precision health to reshape human health and improve the treatment outcomes of breast, lung, and colorectal cancer [4] by providing daily critical data to reduce mortality in patients of all ages and sexes who are afflicted by the current epidemic of chronic diseases related to lifestyle habits [5], [6]. In the real world, lifestyle and environmental factors are difficult to collect for use in analysis because they must be immediately connected and annotated with disease control situations to make the data meaningful [7].

In 2019, the coronavirus (COVID-19) outbreak overwhelmed the healthcare system and caused dramatic loss of life [8]. COVID-19 caused many to have a higher awareness of their own health status and thus pursue self-health management, seeking effective and real-time health management and service platforms. Increasing evidence shows that long-term continuous remote health management can help reduce the health risks caused by the COVID-19 epidemic [9], [10]. The mortality risk of COVID-19 has also been revealed to be related to underlying health conditions, including obesity, panic disorder, and chronic obstructive pulmonary disease (COPD) [11], [12], [13]. These are common chronic diseases in our daily lives, and are the leading cause of disability and death in the world. According to a report from the National Centers for Disease Control and Prevention, 90% of the 3.8 trillion USD annual healthcare expenditure in the United States comes from patients with chronic and mental illnesses [14]. Given the aging population structure worldwide, the management of chronic diseases is bound to become a global health challenge and an economic burden in the foreseeable future [15].

Based on the above points, there is no efficient way to (1) integrate lifestyle, environmental factors, and medical records to provide personalized health recommendation and (2) support multiple chronic disease groups simultaneously. Hence, the purposes of this research were (1) to develop an AI-assisted telecare platform that enables physicians to remotely monitor the situation of patients with chronic diseases and support the data collection of lifestyle and environmental factors from different sources; (2) to develop scalable modular chronic disease prediction models for early prediction of acute exacerbations of chronic diseases using personal lifestyle factors, environmental factors, and medical questionnaires to help patients improve disease control; (3) to construct an appropriate location-based smartphone application to deliver personalized health promotion for patients and achieve the goal of precision health management.

## II. Related Work

### A. Chronic Disease Prediction Models

Several chronic disease prediction models have been developed in recent years. Goto *et al.* proposed an AECOPD (acute exacerbations of chronic obstructive pulmonary disease) model using demographic features, vital signs, and electronic medical records in the emergency department [16]. They found that the use of machine learning improves the ability to predict critical care and hospitalization among emergency patients with COPD exacerbation over the traditional statistical approach with emergency severity index information. Likewise, Peng *et al.* developed a machine learning approach to predict the prognosis of AECOPD hospitalized patients with clinical indicators. They used vital signs, medical history, inflammatory indicators, and decision trees to help respiratory physicians assess the severity of the patient early and improve patient prognosis [17]. Lueken *et al.* collected 59 panic disorder patients and compared brain activation areas before and after specific treatment. Comorbidity status has been predicted using a random undersampling tree and MRI images [18]. Butler *et al.* proposed an early childhood obesity prediction model for predicting obesity in 4- to 5-year-old children, using parental and infant data from the Growing Up in New Zealand (GUiNZ) cohort [19]. Despite the good performance of these prediction models using machine learning algorithms and medical records, they are difficult to implement in real-world situations because patients with chronic disease are not always in the hospital and have real-time medical records. Lifestyle and living environment also affect disease control after a patient is discharged from hospital. Nevertheless, there is no predictive models incorporating lifestyle, living environment and medical questionnaires. Comprehensive data collection may have the potential to achieve better predictive power and provide personalized health promotions to help patients improve their health outcomes.

### B. E-Health and M-Health Systems

Recently, various e-health and m-health applications have been proposed for telemedicine, vital sign monitoring, and health management. In 2017, Clarke *et al.* proposed a remote monitoring platform based on the IEEE 11073 standards for personal health devices. The platform was flexible and extensible, allowing the addition of new medical devices with ZigBee/6LoWPAN modules. They provided a gateway to collect blood pressure, SpO2, blood glucose, and body weight data [20]. In 2018, Yang *et al.* designed an IoT-enabled stroke rehabilitation system to enable telemedicine, which consists of a smart wearable armband, machine learning algorithms, and a 3D printed robot hand [21]. The authors demonstrated the real-time assistance from the system help users to strengthen their motion patterns after stroke. The availability of continuous and real-time data will be a key factor in the development of smart healthcare systems, because stakeholders can use these data to make well-informed decisions [22]. McPadden *et al.* demonstrate that a scalable data science platform can offer the opportunity to access comprehensive health care data for computational health care and precision medicine research [23]. Aida *et al.* develop an m-health application to improve the lifestyle behaviors and health literacy of patients with metabolic syndromes. By visualizing conventional health checkup data and enhancing health education materials, they significantly improve self-efficacy and health outcomes, and maintain weight loss and smoking cessation [24]. In sum, e-health and m-health systems must be flexible and scalable to support various vital signs input and meet different disease care needs and expansion needs. Real-time data visualization would help physicians to quickly understand the patient’s situation and formulate appropriate personalized treatment.

## III. Methods and Service Architecture

**FIGURE 1.**
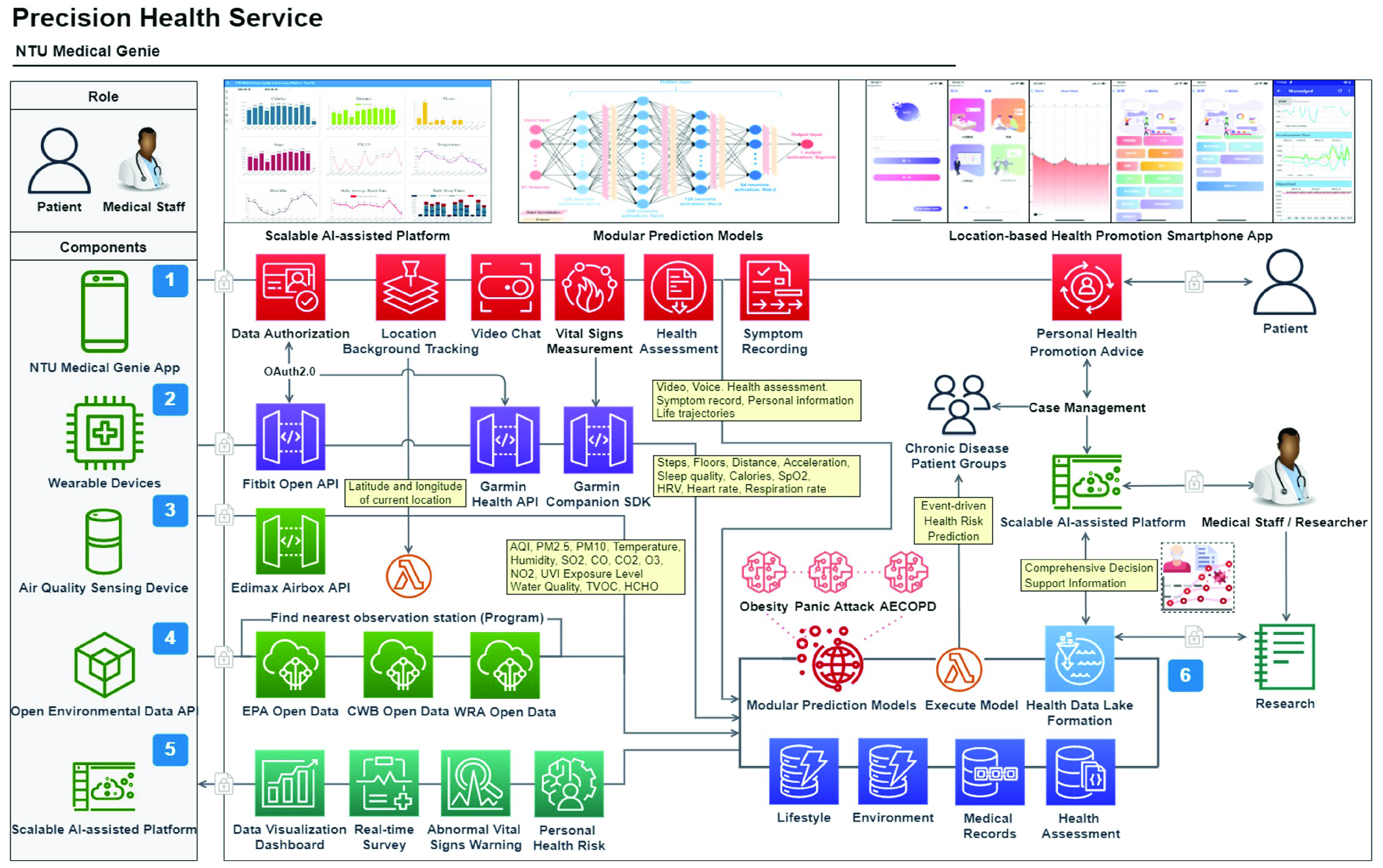
Architecture of precision health service.

The study protocol was approved by the Institutional Review Board of National Taiwan University Hospital (201710066RINB; date of approval: April 19, 2018). Figure 1 shows the architecture of the precision health service. The service consists of the NTU Medical Genie iOS/Android smartphone app, wearable devices, an air quality sensing device, the open environmental data API, the NTU Medical Genie platform, and modular prediction models. After patients are discharged from hospital, all lifestyle and environmental key information would be effectively collected from a wearable device, an air quality sensing device, and a smartphone App. Then, real-time data would be displayed on the platform for medical staff to assist in decision-making. Modular prediction models would be triggered on some very important abnormal vital signs immediately and daily at 2am, ensuring emergency safety and cost-effectiveness. In addition, to achieve high scalability and flexibility, all dataflow nodes such as the number of disease groups, vital signs monitoring devices, or prediction models are designed to run in parallel. So, the nodes could easily be added from platform side or APP side when the load increasing. The corresponding computer resources can be added for stable operation. The following is the detailed description of each component.

### A. NTU Medical Genie Smartphone App

The location-based personal health advice app was developed using Java SE 8 in Android Studio 4.0.1 and Swift 5 in Xcode 12.4. This allowed the measurement of real-time streaming data such as heart rate, heart rate variability, acceleration, SpO2, respiration rate, steps, calorie consumption, and floors climbed via connection with wearable devices. A background location tracking feature and video chat were activated after obtaining user data authorization. For chronic disease management, regular assessment and daily symptom records help physicians understand the patient’s disease condition. As a consequence, this app provided a variety of chronic disease clinical questionnaires and symptom diary functions. These data were automatically uploaded to the server, after which physicians provided personalized health promotion advice in real time through the data visualization platform.

### B. Wearable Devices

To increase the hardware compatibility of this service, wearable devices such as those from Fitbit, Garmin, Apple, Oura Ring, and Asus were included in this study. Real-time lifestyle data (physical activities, heart rate, SpO2, and sleep patterns) were collected and automatically uploaded to a health data lake through the Bluetooth protocol, the open application programming interface, and the software development kit. Time series data were synchronized with the server on average every 15 minutes to ensure that subtle changes were not neglected.

### C. Air Quality Sensing Device

Environmental risks may affect chronic disease control. Patients with chronic respiratory diseases such as chronic obstructive pulmonary disease and asthma are particularly susceptible to air pollution. With the rapid progress of the Internet of Things, home environmental information can be detected by air quality sensors. In this study, an Edimax Airbox was used to collect fine particulate matter (PM2.5) levels, temperature, and humidity at home; these data were uploaded automatically via a wireless network every 15 minutes.

### D. Open Environmental Data API

To better understand the patient’s environmental risks, we used the real-time positioning information obtained by the app and our algorithm to capture the open data from the nearest environmental monitoring station. Data features such as fine particulate matter (PM2.5) levels, air quality index (AQI), sulfur dioxide concentration (SO2), temperature, humidity, UV exposure level, carbon monoxide concentration (CO), water quality, and nitric oxide concentration (NO2) were collected hourly by calling the open data application programming interface from the Environmental Protection Administration, the Central Weather Bureau, and the Water Resources Agency. Historical data was also retrieved by a web crawler, including environmental data from 2011 to 2022.

### E. NTU Medical Genie Platform (Scalable AI-Assisted Telecare Platform)

Through these four information and communication technology methods, comprehensive patient data were collected. To establish an effective connection between patients and physicians, the data platform was designed to provide key information and trend charts to physicians and case managers, facilitating a rapid understanding of the patient’s current condition on one interface and providing patients with personalized health promotion suggestions. In addition to data visualization, this platform provides real-time warning function to assist physicians and case managers in decision making. Physicians and case managers set thresholds for abnormal vital sign warnings according to the patient’s status. When the vital signs exceeded the thresholds, the platform actively triggered the health risks computation process and notified medical staff to intervene if necessary. Regarding the precision health management and prevention of chronic diseases, the platform calculated personal health risks based on modular chronic disease prediction models and the various collected data. Chronic disease prediction models were deployed in online case groups, providing medical staff with optional triggers.

### F. Modular Chronic Disease Prediction Models for Early Prediction of Acute Exacerbation of Chronic Diseases

As mentioned, the health risk value was computed by a robust prediction model and provided as decision support for physicians. The results of chronic diseases such as COPD, panic disorder, and obesity are closely related to the improvement of daily life behavior. Therefore, we implemented these three chronic disease prediction models to demonstrate the scalability of our services. The comprehensive dataset (sections A-D) are pre-processed to extract the key features, followed by the training process. The data pre-processing consists of the last observation carried forward (LOCF) interpolation for inconsistent frequency or null point and re-sampling to deal with the disparate ratio of abnormal event. The normalized data would be trained with a kinds of models and passed an external validation to ensure that models were reliable and applicable to different case groups in the real world. The detailed implementation process of these three models is as follows.

#### 1) Acute Exacerbation of Chronic Obstructive Pulmonary Disease Prediction Model

According to World Health Organization estimates, chronic obstructive pulmonary disease (COPD) will be the third-leading cause of mortality worldwide in 2030 [25]. Acute exacerbations of chronic obstructive pulmonary disease (AECOPD) are associated with substantial morbidity and mortality. Early AECOPD detection will help to reduce mortality. Increasing evidence shows that lifestyle modifications improve efficiency in the self-management and prevention of COPD. Therefore, the aim with our AECOPD prediction model was to use lifestyle data, living environmental data, and clinical questionnaires to predict whether a patient with COPD will experience acute exacerbations of their condition within the next 7 days. The modified Medical Research Council dyspnea scale and the COPD assessment test were used to assess functional impairment and the impact of COPD (cough, sputum, dyspnea, and chest tightness) on health status. All input data features are shown in Textbox 1.

**Environmental features**

Fine particulate matter (PM2.5) levels, air quality index (AQI), sulfur dioxide concentration (SO2), carbon monoxide concentration (CO), and nitric oxide concentration (NO2)

**Lifestyle features**

Heart rate, walking steps, calorie consumption, deep sleep time, light sleep time, rapid eye movement time, awake time

**Clinical questionnaire features**

Chronic obstructive pulmonary disease (COPD) assessment test (9 answers), modified Medical Research Council (mMRC) dyspnea scale (1 answer), life quality questionnaire (5 answers)

Textbox 1. Input data features of AECOPD prediction model

**FIGURE 2.**
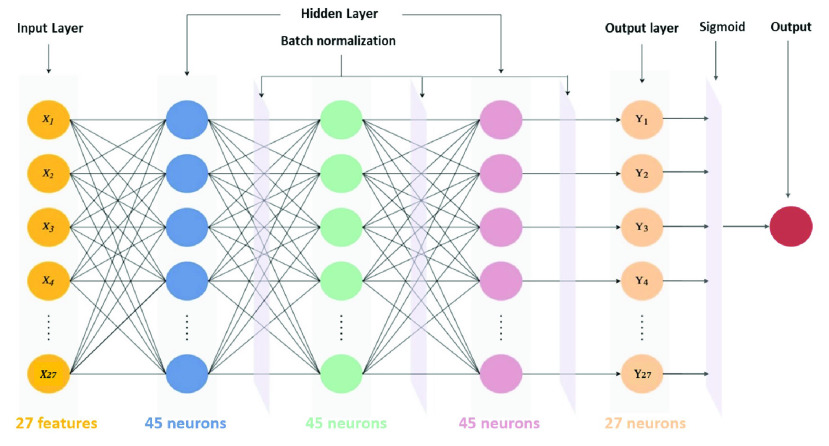
Deep neural network architecture of AECOPD prediction model.

Hyperparameters for machine learning and the deep learning algorithm are presented in Table 1. Decision trees, random forests, linear discriminant analysis, and adaptive boosting were used to implement the AECOPD prediction model. We also propose a deep neural network for comparison with machine learning methods. This was constructed using fully connected layers, which connect each neuron in one layer to every neuron in another layer, mapping feature representations to the target vector space. For the activation function, we used rectified linear units (ReLU) with the introduction of a slope $\alpha $, finishing with the sigmoid function to ensure a probability between 0 and 1. For the optimizer for updating parameters, adaptive momentum estimation (Adam) with quick parameter tuning and rapid convergence was suitable for many parameters. Although Adam used an adaptive learning rate, instead of using its decay function, for this model we used an adaptive learning rate multiplied by 0.1 every 60 epochs. To account for the imbalanced data, we used the class_weights technique from Keras to penalize loss for categorizing data points as the wrong class. The complete deep neural network architecture of the AECOPD model is shown in Fig. 2.

**TABLE 1:** Hyperparameters of AECOPD Models

Model	Parameter	Value
Random forest	n_estimators	300
	min_samples_split	4
	max_depth	30
Decision tree	min_samples_leaf	1
	min_samples_split	2
Linear discriminant analysis	solver	Lsqr
	shrinkage	auto
AdaBoost	n_estimators	45
	learning_rate	1
Deep neural network	hidden layers	(45, 45, 45)
	class_weights	0:10, 1:50
	Adam beta1, beta2	0.7, 0.8

#### 2) Panic Attack Prediction Model

Panic disorder is a kind of anxiety disorder, with a life prevalence of around 2–6% worldwide [26]. A typical panic attack is unexpected and consists of repeated, intense fear attacks, appearing suddenly and reaching a peak within a few minutes. Patients who suffer from panic disorder tend to worry about the occurrence of the next attack and actively try to prevent future attacks by avoiding locations, situations, or behaviors related to the panic attack. Predicting panic attacks accurately may help clinicians to provide timely, appropriate treatment and optimize personalized medicine. Hence, the purpose of this model is to predict whether panic disorder patients will have a panic attack within the next seven days. Random forest, decision tree, linear discriminant analysis, adaptive boosting (AdaBoost), and regularized greedy forest models were implemented to predict panic attacks. The models and hyperparameters are shown in Table 2.

**TABLE 2:** Hyperparameters of Panic Attack Models

Model	Parameter	Value
Random forest	n_estimators	100
	min_samples_split	2
	max_depth	1
Decision tree	min_samples_leaf	1
	min_samples_split	2
Linear discriminant analysis	solver	Lsqr
	shrinkage	auto
Regularized greedy forest	max_leaf	1000
	algorithm	RGF_Sib
	test_interval	100
AdaBoost	n_estimators	50
	learning_rate	1
Deep neural network	fully-connected layer 1	128
	fully-connected layer 2	256
	fully-connected layer 3	128
	fully-connected layer 4	64
	dropout gradient	0.2
	class_weight for negative	1.0
	class_weight for positive	1.5

A deep-learning-based model was also proposed in this study with four fully connected, hidden layers. The activation function in the hidden layers was the rectified linear unit (ReLU), which addressed the problem of disappearing gradients. Batch normalization was applied on each layer after the activation function to accelerate model training and prevent model overfitting. After batch normalization, we also applied dropout to reduce overfitting. We used sigmoid activations for the output layer because we require only a true or false result. The loss function and the optimizer used binary cross entropy (BCE) and Adam, respectively. We selected BCE because the output of the study was binary. BCELoss is defined as Equation 1.

\begin{align*} BCELoss(O,T)=&\frac {1}{n}\sum \limits _{t} (T[i]\ast \log (O[i]) \\&+\,({\textbf {1}} - T[i])*log(1 - O[i]))\end{align*}

Eq. 1. BCELoss equation, where $O$ is the predicted value and $T$ is the ground truth.

**FIGURE 3.**
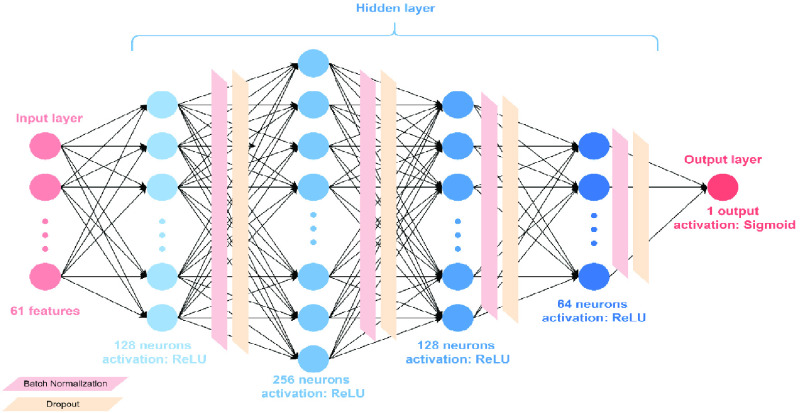
Deep neural network architecture of panic attack model.

We applied an adaptive learning rate (LR) with an initial learning rate of 1e-4. Next, every 50 epochs, we multiplied LR by 0.7. Finally, for increased sensitivity, we accounted for data imbalance using class weights. The principle behind this is to add weight to each category in the training set: low weights are used for categories with many samples. The complete deep neural network architecture is shown in Fig. 3. The input features included continuously measured lifestyle data collected via a wearable device, environmental data obtained from the government’s open data platform, and clinical questionnaire data, as shown in Textbox 2.

**Environmental features**

Fine particulate matter (PM2.5) levels, air quality index (AQI), sulfur dioxide concentration (SO2), carbon monoxide concentration (CO), and nitric oxide concentration (NO2)

**Lifestyle features**

Walking steps, distance, floors, min heart rate, max heart rate, average heart rate, resting heart rate, total sleep duration, deep sleep duration, light sleep duration, REM sleep duration, awake duration

**Clinical questionnaire features**

Beck Depression Inventory (BDI), Beck Anxiety Inventory (BAI), State–Trait Anxiety Inventory (STAI), Panic Disorder Severity Scale (PDSS), Mini International Neuropsychiatric Interview (MINI)

Textbox 2. Input data features of panic attack prediction model

#### 3) Obesity Prediction Model

**FIGURE 4.**
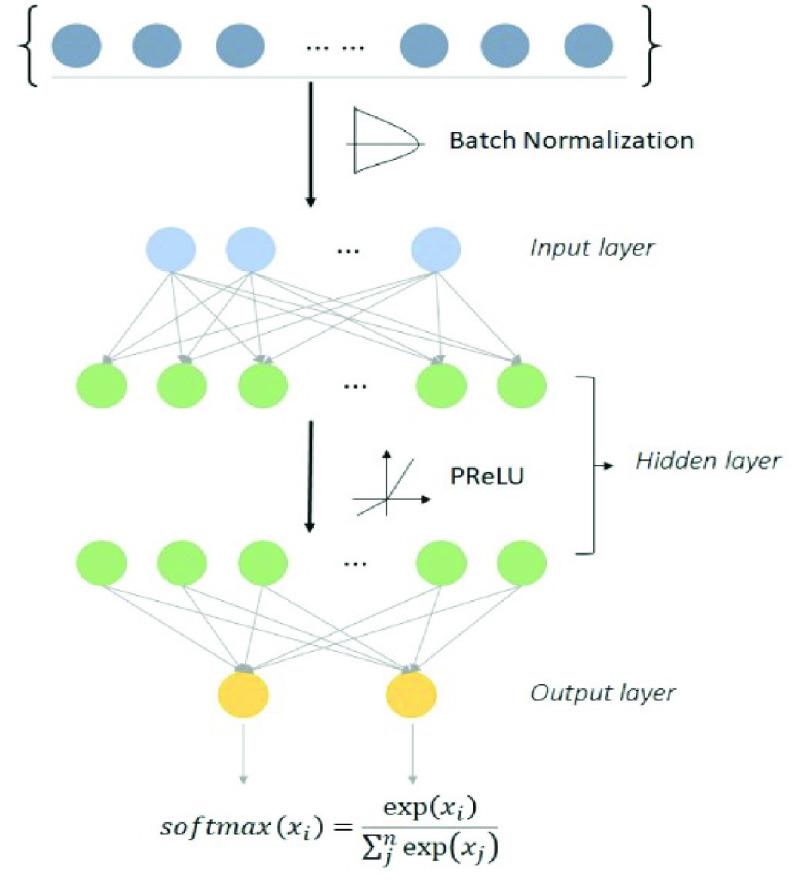
Deep neural network architecture of obesity model.

Evidence indicates that obesity is a common, serious, and costly disease. Obesity cost the US health care system US $\$ $260.6 billion in 2016 [27]. The prevalence of obesity increased from 30.5% to 42.4% from 2010 to 2020. Body mass index (BMI) is a common metric used to screen overweight and obese patients. Increased BMI values are a major risk factor for noncommunicable diseases such as cardiovascular diseases, diabetes, musculoskeletal disorders, and certain cancers. Lifestyle modification and low health literacy are associated with obesity [28], [29]. Hence, the purpose of the obesity model is to predict whether the patient’s BMI will rise within the upcoming 7 days using lifestyle data, environmental data, and health literacy assessment. Machine learning and a deep neural network algorithm were applied to implement the prediction model. The model hyperparameters are presented in Table 3. The deep neural network was constructed using two fully connected layers. Batch normalization and parametric rectified linear units were applied in the process. Figure 4 shows the structure of the DNN model.

**TABLE 3:** Hyperparameters of Obesity Prediction Models

Model	Parameter	Value
Random forest	n_estimators	100
	min_samples_split	2
	max_depth	1
Decision tree	min_samples_leaf	1
	min_samples_split	2
Linear discriminant analysis	solver	Lsqr
	shrinkage	auto
AdaBoost	n_estimators	45
	learning_rate	1
Deep neural network	fully-connected layer 1	128
	fully-connected layer 2	256

#### 4) Validation and Model Assessment

We used 3-fold cross-validation to evaluate the stability of the prediction models. Accuracy, precision, sensitivity, and specificity were used as assessment metrics to evaluate the overall performance, including the closeness and the deviation of the prediction, and the performance on negative and positive cases of the identification models separately based on the validation and test sets. To tune the models for the best performance on the test set, the F1 score was chosen to adjust and evaluate the performance of our multi-feature prediction tasks by varying the outcome thresholds using the validation dataset. Applying the above metrics, our models were well-tuned and evaluated from multiple aspects, yielding high-confidence modular prediction function

#### 5) Feature Engineering and Model Deployment

**FIGURE 5.**
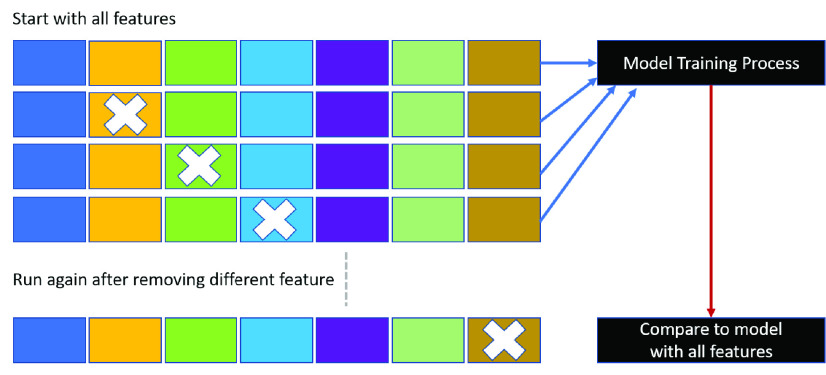
Backward elimination.

Incomplete data is a common issue in real-world apps. To deploy the prediction model in the real world, we implemented the SHAP module (Shapely Additive exPlanations) and a feature selection process to reduce number of variables and the computational cost. The SHAP module was designed to explain the output of prediction models based on cooperative game theory. SHAP module determines the most important features and their influence on the model prediction. The formula of the SHAP value is defined in Equation 2. $\phi i$ is the Shapley value for feature $i$. $S$ is a coalition of features. $p(S)$ is the payoff for this coalition. $N$ is the total number of features. $N/i$ is all the possible coalitions not containing $i$. In this study, a summary plot was applied to describe the distribution and relationship of each feature. Furthermore, feature selection was used to address overfitting and to find the best feature set for a useful, real-world prediction model. We adopted the wrappers method and backward feature elimination to observe the performance change in precision, specificity, and F1 score. We started the model with all features and then removed insignificant features one by one until all features were processed, as shown in Fig. 5. The resulting prediction model with the most cost-effective feature set was deployed on our developed platform.

\begin{equation*} \phi _{i}(p) = \sum \limits _{S\subseteq N/i} x = \frac {|S|!(n - |S| - 1)!}{n!}(p(S\cup i) - p(S))\end{equation*}

Eq. 2. Shapley value calculation

## IV. Results

As of May 25, 2022, the precision health service had served 1,667 patients and 32 medical personnel, derived and monitored 186,986,625 physical data, and performed 6,869 interviews to offer total care to patients. Comprehensive patient information including lifestyle, living environment, life trajectory, disease control, and data on vital signs were collected by a location-based personal health app, open environmental data API, air quality sensing device, and wearable devices that were provided to all participants. All derived data were displayed on the AI-assisted platform and used to train modular prediction models to predict whether a patient with chronic disease would experience acute exacerbation of their condition within the next 7 days.

### A. NTU Medical Genie Platform (Scalable AI-Assisted Telecare Platform)

**FIGURE 6.**
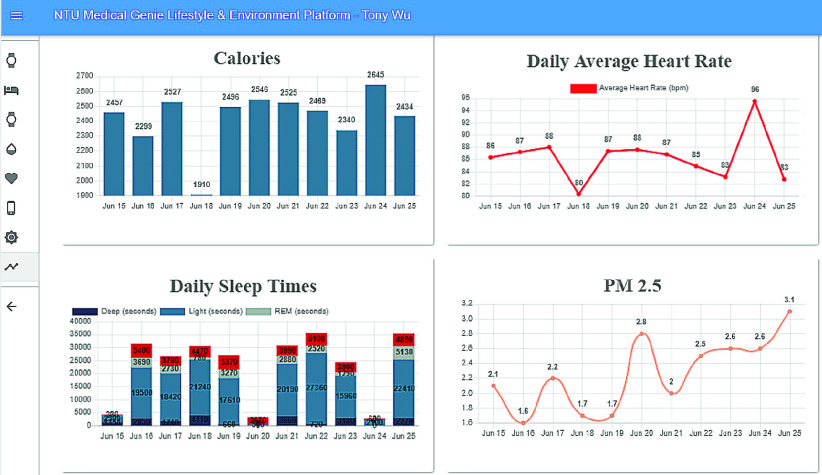
Visualization of lifestyle and environmental data.

An AI-assisted platform for medical staff was developed using the ReactJS frontend framework and the Node.js backend framework. This platform displays the patient’s lifestyle and environmental data trends on a single user interface to help doctors quickly grasp the key information. Fig. 6 shows the overview of our data collection, including both personal lifestyle and environment data.

**FIGURE 7.**
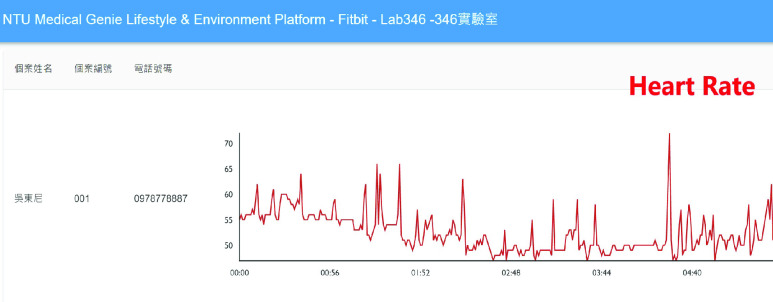
Heart rate graph.

**FIGURE 8.**
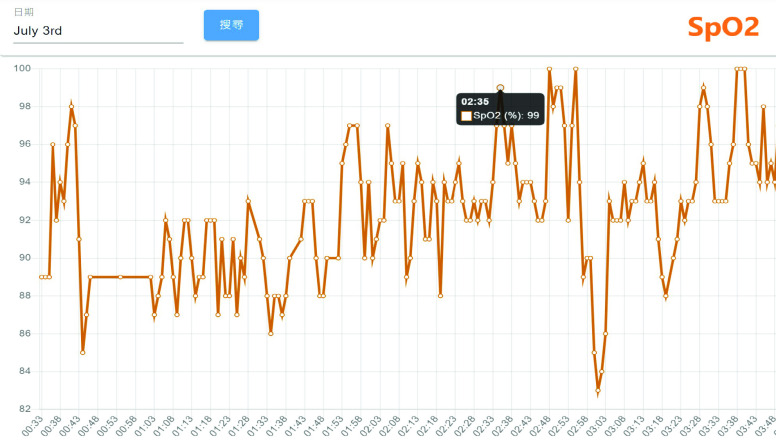
SpO2 trend chart.

**FIGURE 9.**
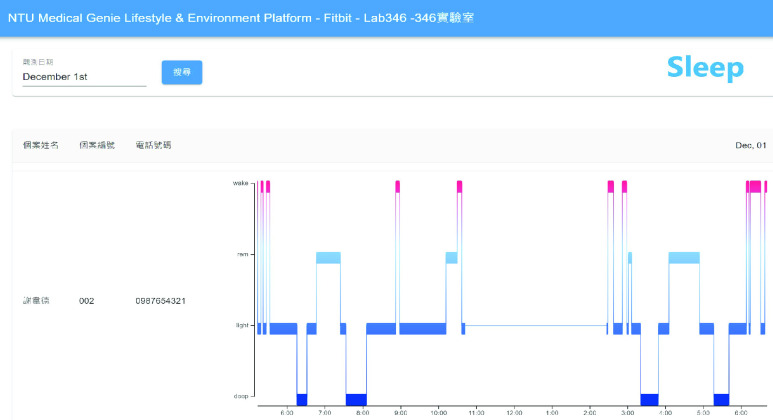
Sleep hypnogram.

**FIGURE 10.**
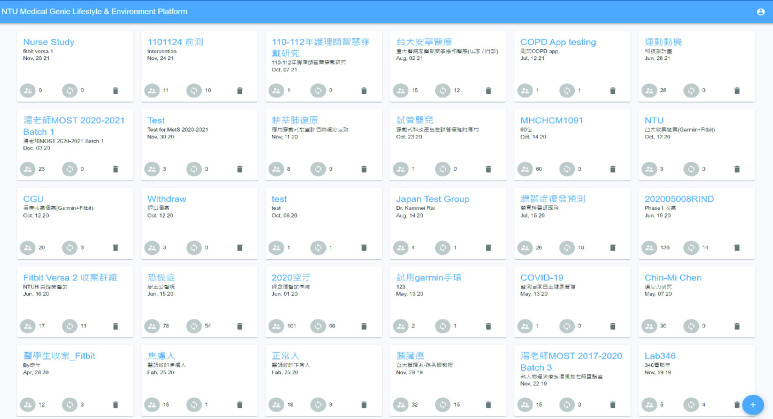
Serve over 30 different chronic disease studies.

**FIGURE 11.**
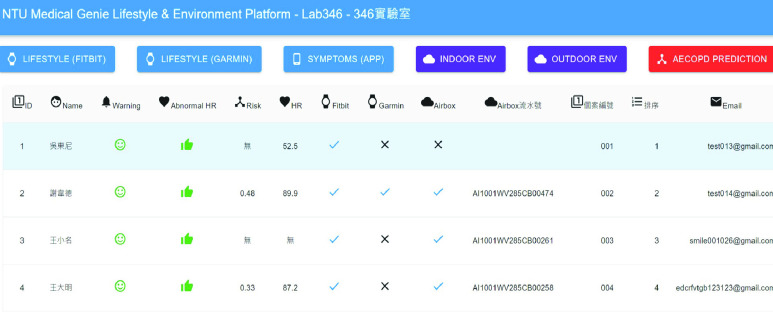
Health risks check list for vital signs.

**FIGURE 12.**
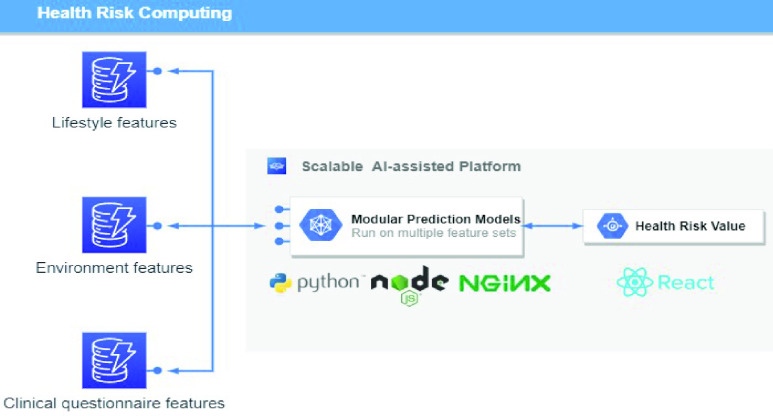
Health risk generation.

Detailed real-time information such as heart rate and SpO2 changes within a few minutes and daily sleep status are viewed by switching to different pages, as shown in Figures 7 and 8. Figure 9 shows that daily sleep status can be divided into four stages: awake, rapid eye movement, light sleep, and deep sleep. This information was collected mainly via wearable devices. To simultaneously support multiple chronic disease healthcare tasks, the platform provides group management functions; modular prediction models were deployed in each group. Physicians created groups to classify patients of different levels, as shown in Fig. 10. Personal health risks were computed by the deployed prediction model. Figure 11 shows an AECOPD health risk scenario: the platform automatically generates today’s health risk based on past data. When the health risk exceeds 0.75, a red icon is displayed to notify the case manager to intervene and care for the patient. Furthermore, the platform allows medical staff to add thresholds for vital signs corresponding to the different patient situations. Patients who exceed a threshold are highlighted by an exclamation mark. After applying this platform in a hospital setting, we found that incomplete data is quite common and critical in the real world. Hence, modular chronic disease prediction models were designed to support prediction of acute exacerbations of chronic diseases via optional features. Figure 12 demonstrates the computation of daily health risk. Even the server receives only lifestyle and environmental data, the health risk is still computed to predict whether abnormal events will occur within the next seven days.

### B. Modular Chronic Disease Prediction Models for Early Prediction of Acute Exacerbation of Chronic Diseases

In this section, we present the proposed modular chronic disease prediction models for generating health risk alerts. Machine and deep learning algorithms were applied to train the prediction model to compute the health risk value by processing lifestyle data, environmental data, and the patient’s medical records. Below are three validated chronic disease prediction models.

#### 1) Acute Exacerbation of Chronic Obstructive Pulmonary Disease Prediction Model

During the study period, we recruited 177 patients diagnosed with COPD according to the Global Initiative for Chronic Obstructive Lung Disease (GOLD) criteria and adult COPD patients who were not implanted with a pacemaker and were not pregnant. To prevent AECOPD earlier and fit diverse scenarios, we implemented multiple models using various combinations of data features to predict acute exacerbations in the next seven days. Table 4 shows the performance of the implemented models on the validation dataset. Compared with the other algorithms, the random forest and deep neural network algorithms yielded the best performance in most indicators.

**TABLE 4:** Performance of Each Model With All Features (AECOPD)

Model	Accuracy	Sensitivity	Specificity	Precision	F1
Random forest	0.914	0.877	0.955	0.955	0.914
Decision tree	0.792	0.712	0.881	0.867	0.782
LDA	0.829	0.781	0.881	0.877	0.826
AdaBoost	0.886	0.822	0.955	0.952	0.882
DNN	0.921	0.904	0.940	0.943	0.923

For 7-day AECOPD prediction, the original AECOPD predictive model achieved an accuracy of 91.4%, a precision of 95.5%, and an F1 score of 91.4% on the validation dataset. To ensure the model applies to the real world, we trained the model with different feature sets and extracted the best performance model for deployment on the platform. Table 5 shows the model performance given different feature sets. Prediction with all features yielded the best performance. Note that the model with only automatically uploaded features also achieved good predictive performance. This model may directly help patients in their daily life. The results further confirm that lifestyle and environmental data features are more important for AECOPD prediction than clinical questionnaire evaluation.

**TABLE 5:** Performance Given Different Feature Sets (AECOPD)

Feature	Model	Accuracy	Sensitivity	Precision	F1
All features	Random forest	0.921	0.904	0.943	0.923
Environmental & lifestyle features	Decision tree	0.835	0.794	0.878	0.834
Clinical questionnaire features	LDA	0.695	0.714	0.769	0.740

**FIGURE 13.**
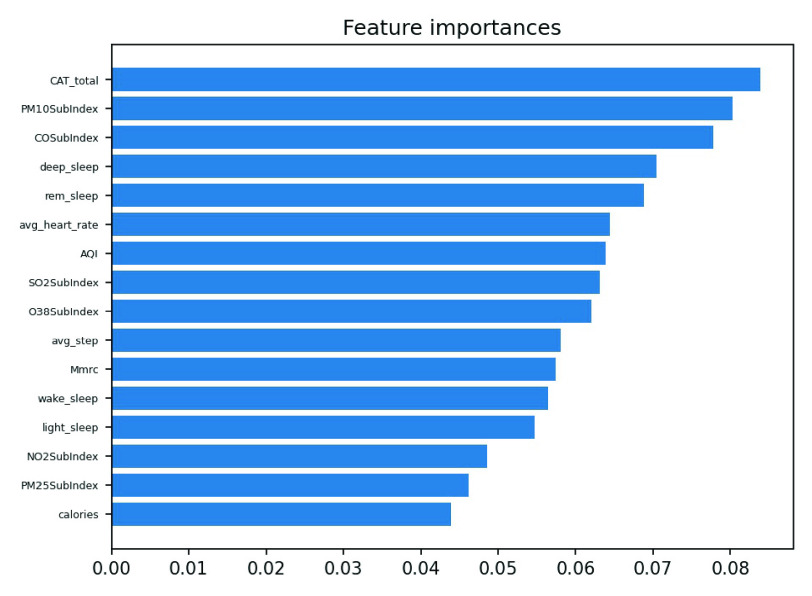
Feature importance scores of AECOPD model.

**FIGURE 14.**
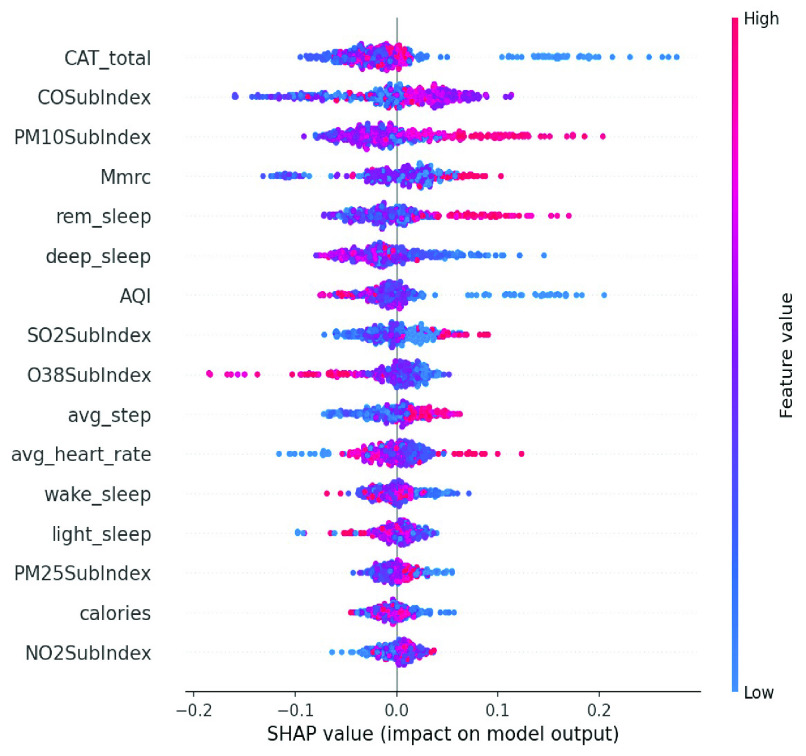
Summary plot of AECOPD model.

**FIGURE 15.**
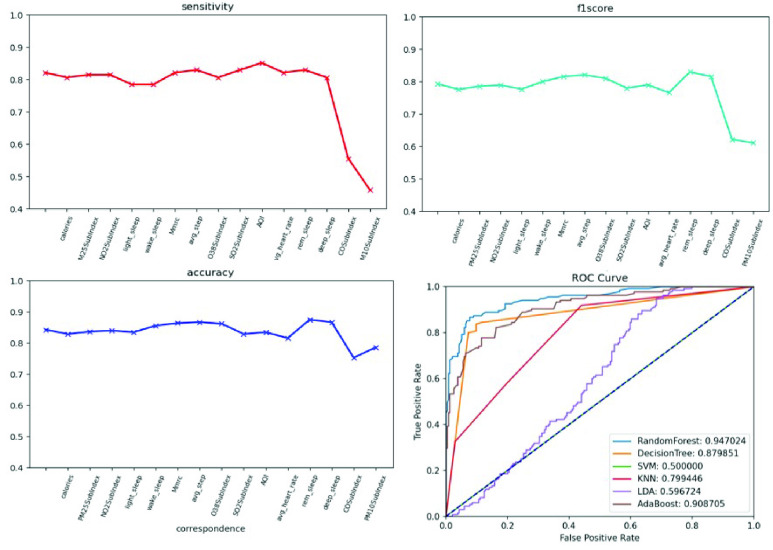
Model performance under feature selection process (AECOPD).

We conducted an external validation to ensure that the model could be applied to other regions. The training dataset and validation datasets were composed of data for 140 patients from National Taiwan University Hospital, and the testing dataset came from 39 patients in Cardinal Tien Hospital. Table 6 demonstrates the model performance on the validation and testing datasets. Random forest, decision tree, and deep neural network were selected as candidates due to their superior results on the validation dataset. Although performance of the AECOPD model declines on the testing dataset, it still achieves an accuracy of 72.4%, a precision of 68.6%, and an F1 score of 68.0%. However, the prediction task requires 27 features to complete the calculation, which is difficult for real-world apps. To reduce the computational costs and number of variables, feature selection and the SHAP module were applied to further analyze the impact of each feature on the prediction model. First, we identified important features affecting the prediction of AECOPD through the feature importance map and SHAP module, as shown in Figures 13 and 14. Then, we implemented backward elimination to compare the performance differences between models without specific features. Figure 15 shows that serious declines in performance occur only when the model does not contain deep sleep time, carbon monoxide concentration (CO), suspended particulate matter (PM10), and total score of COPD assessment test (CAT_total). Hence, we performed the same testing process on the combination of these features to realize the most cost effective prediction model. Table 7 illustrates that the proposed model with the most cost effective feature set achieved superior performance due to the removal of unimportant features. The area under the receiver operating characteristic curve of this model reached 94.7%. In addition, the summary plot also indicated that higher values for features such as the total score of COPD assessment test (CAT_total), air quality index (AQI), and ozone (O3) increase the risk of AECOPD events. Regular exercise (average_step) reduces the risk of AECOPD events.

**TABLE 6:** Model Performance on Validation and Testing Datasets (AECOPD)

Validation	Accuracy	Sensitivity	Specificity	Precision	F1
DNN	0.921	0.904	0.940	0.943	0.923
Random forest	0.914	0.877	0.955	0.955	0.914
Decision tree	0.792	0.712	0.881	0.867	0.782
Testing	Accuracy	Sensitivity	Specificity	Precision	F1
Deep neural network	0.724	0.625	0.783	0.633	0.629
Random forest	0.804	0.689	0.811	0.686	0.680
Decision tree	0.705	0.524	0.814	0.628	0.571

**TABLE 7:** Model Performance After Feature Selection Process (AECOPD)

Feature	Accuracy	Sensitivity	Specificity	Precision	F1
All features	0.804	0.689	0.811	0.686	0.680
Cost-effective features	0.886	0.778	0.949	0.897	0.833

#### 2) Panic Attack Prediction Model

**FIGURE 16.**
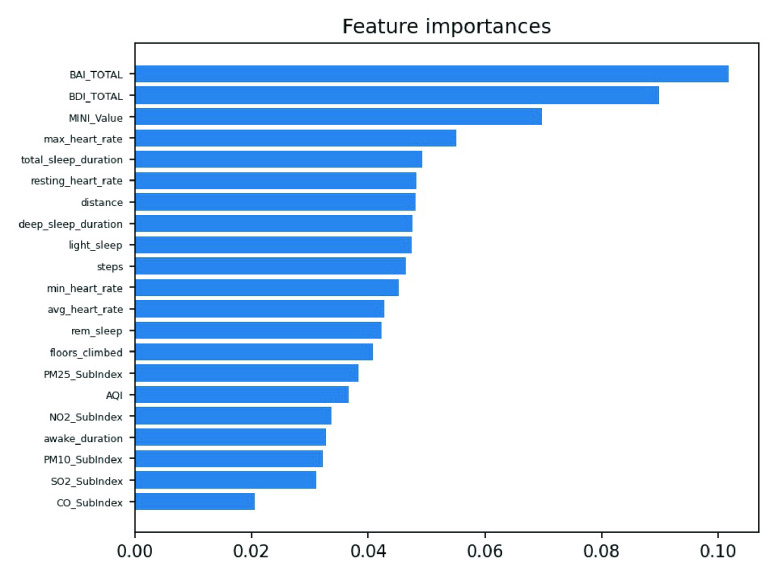
Feature importance scores of panic attack model.

**FIGURE 17.**
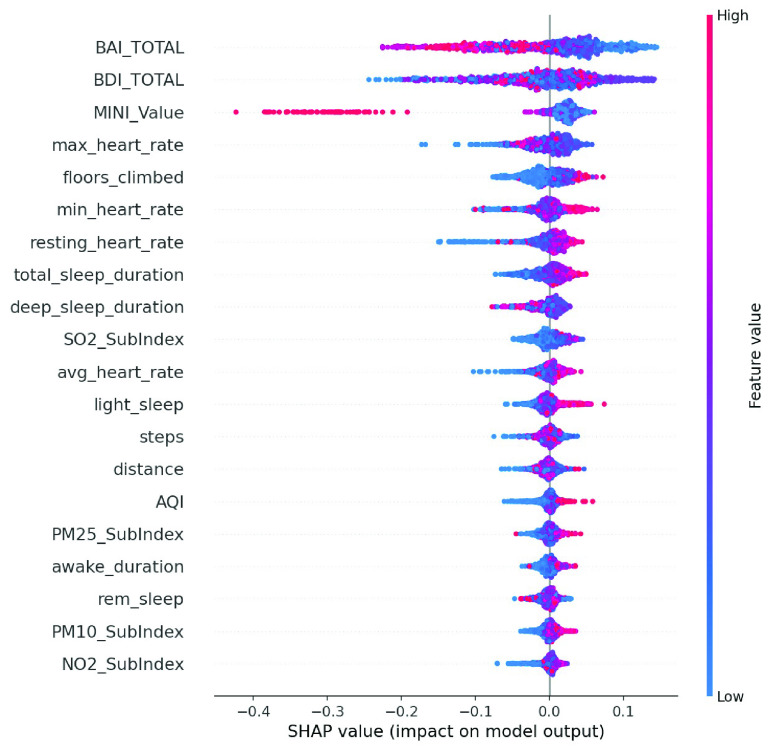
Summary plot of panic attack model.

**FIGURE 18.**
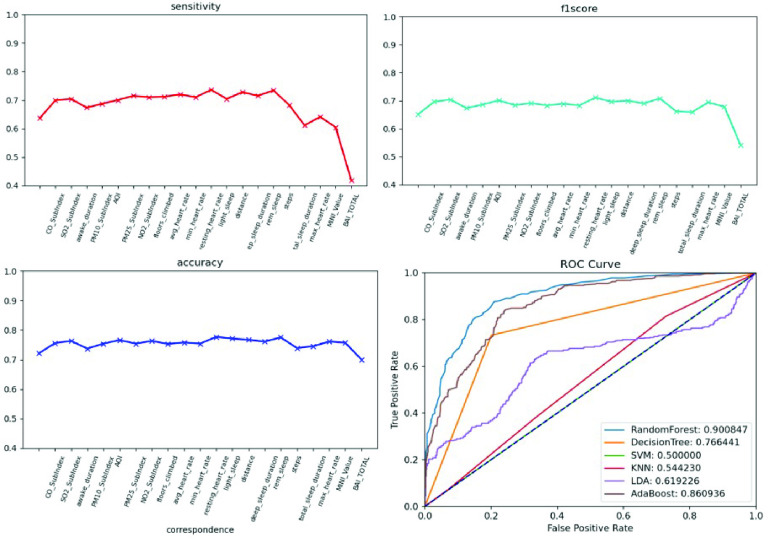
Model performance under feature selection process (panic attack).

We enrolled 70 participants with panic disorder at the En Chu Kong Hospital and MacKay Memorial Hospital. To accurately predict panic attacks, we experimented with deep neural networks and machine-learning classifiers including random forests, decision trees, linear discriminant analysis, adaptive boosting, and regularized greedy forests. Tables 8 and 9 show the model performance on the validation testing dataset. The first 50 patients were included in the training and validation dataset; others were regarded as the testing dataset. The experimental results show the random forest achieved the best performance. However, the sensitivity is worse on the testing dataset. This may reflect data imbalance because the number of panic attacks decreased among patients who were recruited later. To reflect the diversity of real-world scenarios, we trained with different combinations of feature sets, including the all-feature model, the lifestyle-environment model, and the clinical questionnaire model alone, as shown in Table 10. The prediction performance of the all-feature model is better than that of the lifestyle-environment model and the clinical-questionnaire model. However, the all-feature model requires 61 variables to produce predictions, which is difficult to collect in real-world implementations. Therefore, we used the same feature engineering and modules as the AECOPD model to reduce the number of variables and prevent model overfitting. After the feature selection and SHAP process, the feature importance scores and SHAP value are shown in Figures 16 and 17. In the summary plot, data dots to the left of the baseline are prone to panic attack consequences. Data dots to the right of the baseline may serve as health promotion advice to keep patients away from panic attacks. Color represents the data distribution of feature value in our dataset. Raising the values of physical activity features, such as stairs climbed, heart rate, and total sleep time help patients reduce the possibility of panic attacks. Moreover, Fig. 18 shows a severe drop in performance when the model does not include Beck Depression Inventory (BDI_total), Beck Anxiety Inventory (BAI_total), Mini International Neuropsychiatric Interview (MINI_value), and total sleep time. The combination of these features was imported into the same experimental configuration for model training. In Table 11, the cost-effective model achieved an accuracy of 83.1%, a sensitivity of 78.1%, a specificity of 86.1%. and an F1 score of 77.5%. The model requires only four features to yield reliable predictions, which facilitates the real-world deployment of the service.

**TABLE 8:** Validation Dataset Performance With All Features (Panic Attack)

Model	Accuracy	Sensitivity	Specificity	Precision	F1
Random forest	0.975	0.946	0.955	0.992	0.968
Decision tree	0.949	0.931	0.962	0.941	0.936
RGF	0.945	0.896	0.977	0.963	0.928
LDA	0.746	0.583	0.854	0.726	0.647
AdaBoost	0.838	0.772	0.882	0.952	0.792
DNN	0.694	0.602	0.752	0.618	0.612

**TABLE 9:** Model Performance on Validation and Testing Datasets (Panic Attack)

Validation	Accuracy	Sensitivity	Specificity	Precision	F1
Deep neural network	0.694	0.602	0.752	0.618	0.612
Random forest	0.975	0.946	0.955	0.992	0.968
RGF	0.945	0.896	0.977	0.963	0.928
Testing	Accuracy	Sensitivity	Specificity	Precision	F1
Deep neural network	0.722	0.706	0.730	0.576	0.634
Random forest	0.813	0.574	0.938	0.872	0.677
RGF	0.800	0.568	0.920	0.788	0.660

**TABLE 10:** Performance Given Different Feature Sets (Panic Attack)

Feature	Model	Accuracy	Sensitivity	Precision	F1
All features	Random forest	0.975	0.946	0.992	0.968
Environmental & lifestyle features	Regularized greedy forests	0.712	0.483	0.675	0.564
Clinical questionnaire features	Regularized greedy forests	0.748	0.587	0.750	0.659

**TABLE 11:** Model Performance After Feature Selection Process (Panic)

Feature	Accuracy	Sensitivity	Specificity	Precision	F1
All features	0.813	0.574	0.938	0.872	0.677
Cost-effective features	0.831	0.781	0.861	0.769	0.775

#### 3) Obesity Prediction Model

**FIGURE 19.**
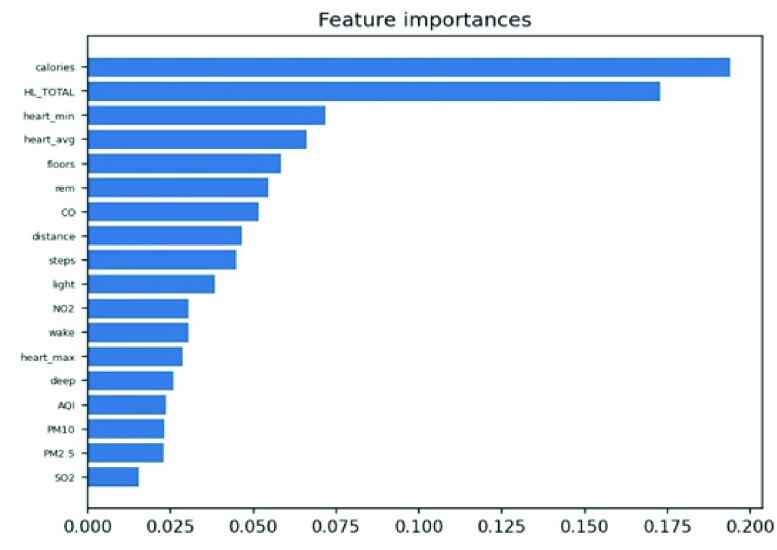
Feature importance scores of obesity model.

**FIGURE 20.**
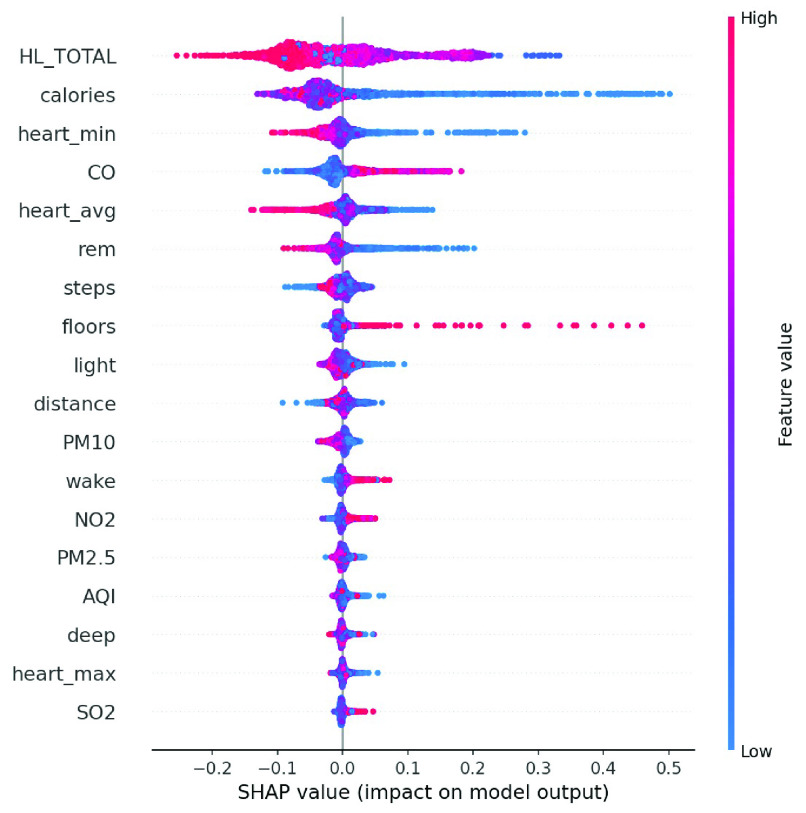
Summary plot of obesity model.

**FIGURE 21.**
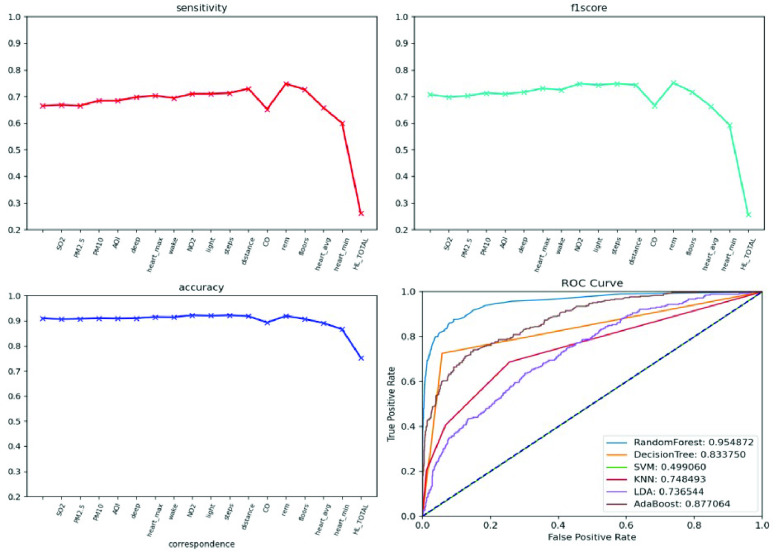
Model performance given feature selection (obesity).

We enrolled 120 obese participants. The main prediction target was whether BMI would worsen within the next seven days. Following the above two methods, we experimented with machine learning methods to train multiple models. Table 12 shows the performance of the proposed models on the validation dataset. The random forest and decision tree achieved better performance. When training the all-feature prediction model, most of the data came from 80 obese patients in central and southern Taiwan. Table 13 shows the performance given different feature sets. As lifestyle and living environment may be very similar, we used the data of 40 obesity patients from northern Taiwan for model-external validation. Table 14 shows that the decision tree achieved the best performance on the testing dataset, albeit with low sensitivity, perhaps due to the significant difference between patients from the north and those from the south. Therefore, we applied the above SHAP module and feature selection to identify the most cost-effective model. Figures 19 and 20 show the distribution of lifestyle factors, living environment, and health literacy data. The results demonstrate that lower values for features such as health literacy, consumption in calories, average heart rate, and rapid eye movement time increase the risk of becoming overweight and obese. Figure 21 shows that serious declines in performance occur only when the model does not contain consumption in calories, health literacy total score, average heart rate, and minimum heart rate. Therefore, the combination of these four features may be the most influential and cost-effective feature set. We executed the same model training and testing process on this feature set. Table 15 illustrates that the proposed model achieved good performance even with a large reduction in features. Moreover, the sensitivity is significantly improved due to the removal of unimportant features. In summary, feature selection facilitated the construction of the most cost-effective predictive model requiring only four features. For 7-day BMI prediction, the proposed predictive model with cost-effective features achieved an accuracy of 93.7%, a sensitivity of 71.0%, a specificity of 98.1%, and an F1 score of 78.6% on the testing dataset. This model is suitable for deployment on the platform and has the potential to yield reliable predictions of future obesity events.

**TABLE 12:** Model Performance With All Features (Obesity)

Model	Accuracy	Sensitivity	Specificity	Precision	F1
Random forest	0.967	0.906	0.992	0.978	0.941
Decision tree	0.953	0.908	0.971	0.927	0.918
LDA	0.834	0.572	0.940	0.793	0.665
AdaBoost	0.867	0.707	0.931	0.806	0.753
DNN	0.886	0.734	0.942	0.823	0.776

**TABLE 13:** Performance Given Different Feature Sets (Obesity)

Feature	Model	Accuracy	Sensitivity	Precision	F1
All features	Random forest	0.967	0.906	0.978	0.941
Environmental & lifestyle features	Decision tree	0.940	0.882	0.882	0.882
Clinical questionnaire features	Decision tree	0.666	0.357	0.416	0.384

**TABLE 14:** Model Performance on Validation and Testing Datasets (Obesity)

Validation	Accuracy	Sensitivity	Specificity	Precision	F1
DNN	0.886	0.734	0.942	0.823	0.776
Random forest	0.967	0.906	0.992	0.978	0.941
Decision tree	0.953	0.908	0.971	0.927	0.918
Testing	Accuracy	Sensitivity	Specificity	Precision	F1
DNN	0.910	0.471	0.978	0.772	0.585
Random forest	0.931	0.503	0.998	0.978	0.665
Decision tree	0.917	0.697	0.951	0.689	0.693

**TABLE 15:** Model Performance After Feature Selection (Obesity)

Feature	Accuracy	Sensitivity	Specificity	Precision	F1
All features	0.917	0.697	0.951	0.689	0.693
Cost-effective features	0.937	0.710	0.981	0.880	0.786

### C. Location-Based Smartphone Application to Deliver Real-Time Personalized Health Promotion for Patients with Chronic Diseases

**FIGURE 22.**
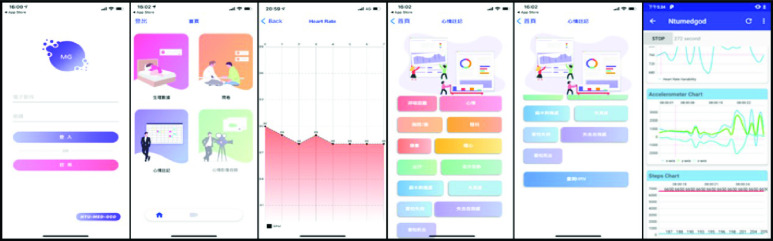
Smartphone application interface.

To obtain real-time information and provide a health promotion service, we developed a location-based smartphone application for the Android and iOS operating systems. The application interface is shown in Fig. 22. First, the patient registered an account in the app. The information to be input included the name, birthday, phone number, attending physician, and so on. All relevant patient registration information was stored in a firebase. A background location tracking feature was activated after the user authorized location data. The collected real-time latitude and longitude data was converted into parameters for calling the open environmental data API to calculate the environmental exposure risk for the user’s location. The content of the app varied depending on the type of chronic disease. For example, patients with panic disorder were presented with four main functions when entering the homepage: real-time physiological data measurement, a self-evaluation questionnaire, symptom recording, and video chat. On the physiological data measurement page, real-time physiological data to be collected include heart rate, SpO2, heart rate variability, and acceleration. When the user successfully entered the physiological data monitoring page, the data trend graph started, and the data sampling rate was changed to once per second for upload to the InfluxDB time series database. On the self-evaluation questionnaire page, panic attack related clinical questionnaires including PDSS, STAI-S, and STAI-A were provided as online surveys for users to fill out to reflect their own health conditions. In addition, 21 symptom buttons were provided on the symptom recording page to record the exact time and accurately mark the significance of lifestyle and living environmental data for disease control. The time of discomfort and symptoms were then sent to the database. At the same time, the heart rate variability (HRV) measurement function and video chat function both were activated, and the HRV value and symptom records were transmitted to the AI-assisted platform so that psychiatrists and case managers could offer timely and appropriate personalized health promotion advice based on their situation. This app has been used in many hospitals (National Taiwan University Hospital, En Chu Kong Hospital, Fu Jen Catholic University Hospital, Tri-Service General Hospital, Cardinal Tien Hospital, MacKay Memorial Hospital, and Okayama University Hospital) in Taiwan and Japan. To protect user privacy, the app was designed to transmit data via the https protocol, and personal information was encrypted. Case managers could instantly ensure the sustainability of data via the AI-assisted platform and provide users with appropriate health advice on disease control and lifestyle modification.

## V. Conclusion

We designed and implemented a scalable precision health service for patients with chronic diseases. The results demonstrate that this service provides continuous monitoring of lifestyle and environment, instant warnings in the event of abnormal vital signs, and decision support based on modular predictive models. Compared with existing studies, we have created an unprecedented new service and improved the performance of chronic prediction models by applying objective lifestyle and environmental factors. At the same time, we have used feature engineering to reduce the computational costs and enhance the practicality of real-world AI prediction models. The proposed prediction models require a small number of features to achieve excellent performance in predicting whether a patient with chronic disease will experience an abnormal event within the next 7 days. Furthermore, we address the inability to quantify and extract lifestyle and environmental information in past studies by integrating wearable devices, open data, indoor air quality sensors, smartphone applications, and a healthcare platform. To the best of our knowledge, this is the first study to use continuous lifestyle factors, environmental factors, clinical factors, feature selection, and artificial intelligence to predict abnormal events in chronic diseases and deploy to the real world with external validation.

As of May 25, 2022, the precision health service had served 1,667 patients and 32 medical personnel in Taiwan and Japan, derived and monitored 186,986,625 physical data, and conducted 6,869 interviews to offer total care to patients. The parallel operation of system dataflows can improve scalability and flexibility, and is not limited by a single process or device control, which can support the increase of different care needs in the future. It has the potential to become the next-generation e-health system to assist physicians in remote care and establish an effective communication channel between medical personnel and patients. Traditionally, patients with chronic diseases must return to the hospital periodically for numerous clinical tests to observe their health condition. They may run the risk of acute exacerbation between routine visits. However, with the proposed service, all chronic disease related data are uploaded automatically, including questionnaire assessments and lifestyle and environmental information. The patient’s health risk value is computed through modular predictive models, reminding patients and medical personnel in advance to improve their health outcome. The resultant comprehensive view of patient data could help physicians and patients to formulate personalized health promotion plans and achieve precision health management. Our results also confirm that lifestyle and environmental data are highly correlated to patient health conditions, and have a strong influence on the early warning of acute exacerbations. By applying the SHAP module and feature engineering, we clearly identify the impact of physical activity, sleep quality, and heart rate on chronic disease control, and provide precise recommendations for health improvements for physicians and patients. In the future, we will strengthen the precision health service to support more data collection for lifestyle factors, and implement digital twin models [30] to further automatically provide concrete health promotion advice for patients with chronic diseases.
